# Genetic analysis of an F_2 _intercross between two chicken lines divergently selected for body-weight

**DOI:** 10.1186/1471-2164-10-248

**Published:** 2009-05-27

**Authors:** Per Wahlberg, Örjan Carlborg, Mario Foglio, Xavier Tordoir, Ann-Christine Syvänen, Mark Lathrop, Ivo G Gut, Paul B Siegel, Leif Andersson

**Affiliations:** 1 Department of Medical Biochemistry and Microbiology, Uppsala University, BMC, BOX 597, S-75124 Uppsala, Sweden; 2 Department of Animal Breeding and Genetics, Swedish University of Agricultural Sciences, BMC, BOX 597, S-75124 Uppsala, Sweden; 3 CEA/IG-Centre National de Génotypage, 2 rue Gaston Crémieux, 91057 Evry Cedex, France; 4 Molecular Medicine, Department of Medical Sciences, Uppsala University, Uppsala, Sweden; 5 Virginia Polytechnic Institute and State University, Department of Animal and Poultry Sciences, Blacksburg, VA 24061-0306, USA; 6 Génomique animale GIGA-R, avenue de l'hôpital 1, B-4000 Liège, Belgium

## Abstract

**Background:**

We have performed Quantitative Trait Loci (QTL) analysis of an F_2 _intercross between two chicken lines divergently selected for juvenile body-weight. In a previous study 13 identified loci with effects on body-weight, only explained a small proportion of the large variation in the F_2 _population. Epistatic interaction analysis however, indicated that a network of interacting loci with large effect contributed to the difference in body-weight of the parental lines. This previous analysis was, however, based on a sparse microsatellite linkage map and the limited coverage could have affected the main conclusions. Here we present a revised QTL analysis based on a high-density linkage map that provided a more complete coverage of the chicken genome. Furthermore, we utilized genotype data from ~13,000 SNPs to search the genome for potential selective sweeps that have occurred in the selected lines.

**Results:**

We constructed a linkage map comprising 434 genetic markers, covering 31 chromosomes but leaving seven microchromosomes uncovered. The analysis showed that seven regions harbor QTL that influence growth. The pair-wise interaction analysis identified 15 unique QTL pairs and notable is that nine of those involved interactions with a locus on chromosome 7, forming a network of interacting loci. The analysis of ~13,000 SNPs showed that a substantial proportion of the genetic variation present in the founder population has been lost in either of the two selected lines since ~60% of the SNPs polymorphic among lines showed fixation in one of the lines. With the current marker coverage and QTL map resolution we did not observe clear signs of selective sweeps within QTL intervals.

**Conclusion:**

The results from the QTL analysis using the new improved linkage map are to a large extent in concordance with our previous analysis of this pedigree. The difference in body-weight between the parental chicken lines is caused by many QTL each with a small individual effect. Although the increased chromosomal marker coverage did not lead to the identification of additional QTL, we were able to refine the localization of QTL. The importance of epistatic interaction as a mechanism contributing significantly to the remarkable selection response was further strengthened because additional pairs of interacting loci were detected with the improved map.

## Background

This study is based on two unique chicken lines that have been developed by bi-directional selection for body-weight at 56 days of age [1]. The two lines originated from the same base population and 40 generations of divergent selection resulted in more than an eight-fold difference in body-weight at the age of selection between the High-Weight (HWS) and the Low-Weight selected (LWS) lines. We have bred a large F_2 _intercross between these lines as a resource population for QTL analysis [2,3]. Despite a powerful experimental design (~850 F_2_) a previous QTL analysis could only explain about 13% of the residual phenotypic variation for body-weight at selection age in the F_2 _population and at most 50% of the difference between the parental lines [2]. These results indicated that the phenotypic difference between the two chicken lines may be attributed to many QTL each with small individual effects in concordance with the infinitesimal model for polygenic inheritance. We identified 13 QTL (denoted *Growth1 *– *Growth13*) and at each locus the allele from the HWS line was associated with increased growth consistent with the line difference [2].

To further study the genetic architecture causing the phenotypic difference between the HWS and LWS lines Carlborg et al. [4] analyzed the QTL data using a genetic model testing for pair-wise interaction between loci (epistasis). The epistatic analysis revealed a network of six interacting loci, all interactions involving the *Growth9 *QTL on chromosome 7. *Growth9 *was also detected as the only QTL reaching genome-wide significance in the standard QTL analysis of body-weight at 56 days. The growth-promoting effect of the interacting loci was highly dependent on the genotype at *Growth9 *as HWS homozygosity at this locus was required for the other interacting loci to have a significant effect on growth. Moreover, LWS line homozygosity at either *Growth9 *or all three of the loci interacting with *Growth9 *effectively led to the network having no effect on growth. Hence, the significant marginal effect observed in the one-locus QTL analysis for *Growth9 *and the suggestive evidence for marginal effects of the three other major loci in the network is manifested solely by the interactions between *Growth9 *and the other loci in the network. The genetic network was estimated to account for about 45% of parental line difference at 56 days of age [4].

The previous analyses [2-4] were carried out using a rather sparse microsatellite-based linkage map. The map lacked markers on 13 microchromosomes and there were also several regions with poor coverage. Therefore, it was possible that important QTL were missed due to lack of markers. A high-density linkage map would increase the power to detect QTL and enhance the precision in the QTL analysis.

Here we report results from a QTL analysis, based on a comprehensive linkage map, for body-weight traits using our intercross between the HWS and LWS lines. First, we performed a standard genome-wide one-dimensional QTL analysis to fit a one-locus model to detect QTL with main effects. Secondly, we performed a genome-wide pair-wise search to detect epistatic interactions between loci. Finally, we used data on about 13,000 SNPs to compare allele frequency distributions between the HWS and LWS lines to investigate if we could detect any molecular footprints in the form of selective sweeps caused by selection for body-weight.

## Methods

### Chicken lines and intercross

A large F_2 _intercross pedigree was set-up by crossing individuals from the HWS and LWS lines [2,3]. The two parental lines originated from a common base population formed by intercrossing seven partially inbred White Plymouth Rock lines of chicken. The selected lines have been developed and maintained at Virginia Polytechnic Institute and State University, Blacksburg, VA. The sex-average mean body-weight, at the age of selection, for the HWS line was at generation forty 1522 g (SD: ± 36), compared to the LWS line with a sex-average mean of 181 g (SD: ± 5.4). Comprehensive information regarding the development, maintenance and characteristics of the selection lines have been previously reviewed by Dunnington and Siegel [1].

The development of the intercross and a detailed description of the husbandry have previously been described [3]. Briefly, to produce the reciprocal intercross (i.e., HL and LH F_2 _progeny) we mated 10 HWS line males with 22 LWS line females and eight LWS line males to 19 HWS line females. From the F_1 _generation four HL males were mated to 37 LH females and, four LH males were mated to 38 HL females to produce an F_2 _population consisting of 874 chickens. The F_1 _and F_2 _animals were housed in the same facilities and fed the same dietary as the parental selection lines. Chickens were provided *ad libitum *access to water and feed. The F_2 _generation was produced in a single hatch and all animals were sacrificed at 70 days of age.

### Phenotypic traits

Table 1 summarizes the phenotypic traits analyzed in this study. Body-weight measurements were recorded for each F_2 _bird at hatch, at 14, 28, 42, 56 and at 70 days of age.

**Table 1:** Phenotypic traits analyzed in the F_2 _intercross.

Trait^1^	n	Mean ± SD	Range^2^
**Body-weight (g)**			
14 days	874	75.2 ± 14.9	38 – 144
28 days	871	179.1 ± 56.8	53 – 385
42 days	809	365.5 ± 113.1	76 – 729
56 days	795	621.6 ± 186.9	134 – 1179
70 days	789	943.3 ± 262.1	182 – 1627
**Growth (g)**			
0–14 days	874	47.4 ± 14.7	12 – 116
14–28 days	871	103.8 ± 47.3	-10 – 241
28–42 days	809	179.5 ± 68.1	0 – 372
42–56 days	794	251.7 ± 88.6	-103 – 466
56–70 days	788	320.7 ± 94.9	-82 – 778

Sex-average mean, standard deviation (SD), number of individuals (n) and the phenotypic range are given for each analyzed body-weight and growth trait.

^1^Body-weight was measured at six different ages starting from hatch to 70 days. Growth traits were calculated as the differences between body-weights at the two weeks interval.

^2^Negative values for growth traits occur because some individuals lost weight during a two-week interval.

### DNA isolation, marker selection and genotyping

DNA was extracted from blood by AGOWA (Berlin, Germany) from chickens included in the intercross pedigree (P, F_1 _and F_2_). Methods for SNP selection and the genotyping protocol used to generate the genotypes for ~13,000 SNP markers for 15 individuals from each parental strain have been published elsewhere [5]. A set of 384 highly informative SNPs were selected from the 13,000 SNPs, to enable the construction of a linkage map comprising about one marker every ten cM throughout the chicken genome. SNP genotyping was conducted with the GoldenGate assay (Illumina, CA) at a DNA concentration of 50 ng/ul. Animals in the pedigree were genotyped at the SNP technology platform in Uppsala (Sweden) while the ~13,000 SNP panel was analyzed at CNG in Paris (France).

### Construction of linkage maps

We used the CRI-MAP software package to construct linkage maps [6]. We have previously reported a microsatellite-based linkage map constructed using this intercross pedigree [7]. To build the current autosomal linkage map, genotype data from 138 microsatellites were merged with data from 351 SNPs. The dataset was checked for non-Mendelian inheritance errors using the *prepare *option in CRI-MAP. Markers were then ordered according to the May 2006 genome assembly and the *fixed *option in CRI-MAP was used to estimate sex-average map distances, in Kosambi cM, between loci. Markers that were not included in the genome assembly were incorporated at the most likely position in the linkage map based on results from a two-point linkage analysis. The order of markers was evaluated using the functions *flips *and *chrompic *in CRI-MAP.

### Statistical analysis

Factors affecting phenotypic trait variation in the F_2 _population were identified using analysis of variance (ANOVA). Sex was included as a fixed effect in the QTL model for all body-weight traits. In contrary to the analysis performed by Park et al. [3] and Jacobsson et al. [2] using the same F_2 _intercross, but consistent with our previous analysis of epistatic interaction [4] we did not include family (F_2 _full sibling family) as a fixed effect in our QTL model because the number of full-sib families was large (75 in total) and because maternal effects in chicken are expected to be small. Furthermore, we did not include reciprocal mating in the QTL model because our previous analysis showed that mating type did not have a significant effect on growth [3].

The QTL analysis of autosomes was performed using interval mapping as first described by Lander and Botstein [8] and later modified for analyzing crosses between outbred lines by Haley and co-workers [9,10]. We used QTLExpress [11] to calculate QTL genotype probabilities conditional on marker genotypes. These were then used in a least square regression-based QTL analysis conducted using an in-house QTL mapping software [12]. Empirical significance thresholds levels were established for each trait by permutation [13] and the same significance thresholds were used for all traits as the variation between different traits were minimal. The 5% genome-wide significance level was set to an F-value > 9 and as genome-wide suggestive level we used the chromosome-wide significance level for chromosome 4 (F-value > 6), because this chromosome represents approximately 5% of the chicken linkage map. With this suggestive level of significance we expect to find one false positive QTL (type-1 error) per trait and genome scan. Epistatic interaction analysis was conducted as described by Carlborg et al. [4,12]. In short, a statistical model including sex as a fixed effect, the additive, dominance and all pair-wise epistatic effects (additive-by-additive, additive-by-dominance, dominance-by-additive and dominance-by-dominance) of QTL pairs was used. This epistatic analysis increases the power to identify loci whose effect is dependent on the genotype at another locus. QTL pairs that reached the 5% genome-wide significance threshold in a randomization test for the joint effect of the epistatic pair (no QTL vs. two interacting QTL or one QTL with significant individual effect vs. two interacting QTL) and a 1% significance threshold in a model-selection randomization test for the joint effect of the epistatic parameters (two non-interacting QTL vs. two interacting QTL) are reported as epistatic pairs. The randomization testing procedure was previously described in detail [14]. QTL peaks located within 25 cM of each other were assumed to represent the same locus.

To visualize the effect of individual QTL pairs and also to estimate the total contribution of the epistatic network, we produced a dataset including only F_2 _individuals where the genotype for all QTL in the main four locus network affecting body-weight at 56 days of age could be determined with high confidence (n = 727). Only individuals where the genotype probabilities exceeded 0.70 for all four loci in the network were selected. The individuals were then classified according to the most probable genotype (i.e. HH, HL or LL) for each locus. We calculated sex-averaged phenotypic means for all nine two-locus genotype classes and plotted the data. The combined effect of the epistatic loci was visualized as follows. First, the dataset was stratified according to the genotype at the main locus *Growth9*, one group to include the HWS homozygotes, a second group to contain the heterozygotes and a third group including LWS homozygotes individuals (HH; n = 176, HL; n = 365, LL; n = 186). For each of the three groups we then created four more strata with individuals that were HWS homozygous for 0, 1, 2 or 3 of the loci *Growth4*, *Growth6 *and *Growth12*. A one-sided two-sample t-test was used to test for differences in mean body-weight at 56 days of age between the groups.

Fst values [15] were calculated for each of the segregating SNP markers using the GenePop software (version 4.0) [16] to estimate the degree of differentiation between lines because the two lines are expected to show marked allele frequency differences at those loci that have responded to the divergent selection. Observed homozygosity was measured as the number of homozygous individuals divided by the total number of individuals. Confidence intervals for QTL regions for the body-weight trait with the highest statistical significance were defined with the one-LOD drop method [17].

## Results

### A linkage map including 434 genetic markers

We designed Illumina assays for parallel genotyping of 384 SNP markers segregating in the HWS/LWS intercross in order to establish a linkage map with an improved genome coverage compared to our previous microsatellite map. Table 2 summarizes the linkage map comprising altogether 316 SNPs and 118 previously genotyped microsatellites [7]. In Additional File 1 the marker order together with the positions in cM and in Mb are presented.

**Table 2:** Summary of the linkage map.

	Markers							
						
Chromosome	SNPs	Microsats	Total	Map distance (cM)	Physical coverage (Mb)^1^	Chr. length (Mb)^2^	%Coverage	cM/Mb
1	58	16	74	497.2	196.0	201.0	97.5	2.5
2	42	16	58	332.8	152.5	154.9	98.0	2.2
3	23	9	32	262.5	104.7	113.7	92.1	2.5
4	25	11	36	210.6	90.7	94.2	96.3	2.3
5	17	6	23	164.1	59.7	62.2	96.0	2.6
6	8	5	13	69.7	29.6	37.4	79.1	2.4
7	13	4	17	120.3	36.5	38.4	95.1	3.3
8	7	3	10	100.2	26.1	30.7	85.0	3.8
9	7	4	11	135.5	23.1	25.6	90.2	5.9
10	7	7	14	84.3	20.7	22.6	92.0	4.1
11	8	3	11	68.5	19.7	21.9	90.0	3.5
12	7	4	11	81.7	18.0	20.5	87.8	4.5
13	8	5	13	73.9	17.7	18.9	93.7	4.2
14	4	3	7	74.5	14.7	15.8	93.0	5.1
15	6	3	9	53.1	10.0	13.0	77.0	5.3
16_1^3^	1	-	-	-	-	-	-	-
16_2^3^	2	-	-	-	-	-	-	-
17	6	2	8	54.8	9.4	11.2	84.0	5.8
18	5	2	7	45.1	8.0	10.9	73.4	5.6
19	8	-	8	46.7	8.7	9.9	87.9	5.4
20	10	2	12	71.7	13.5	14.0	96.4	5.3
21	6	-	6	61.4	6.2	7.0	88.6	9.9
22	2	-	2	2.7	0.4	3.9	10.3	6.8
23	5	-	5	49.6	5.4	6.0	93.3	9.2
24	5	2	7	51.3	5.6	6.4	86.9	9.2
25	6	-	6	53.1	1.9	2.0	93.6	27.9
26	4	4	8	52.8	4.3	5.1	84.3	12.3
27	6	3	9	59.1	3.8	4.8	79.2	15.6
28	4	1	5	51.7	3.4	4.5	75.6	15.2
LG1^4^	5	2	7	25.5	-	-	-	-
GCT004^5^	-	1	1	-	-	-	-	-
rs16748775^5^	1	-	1	-	-	-	-	-

Total	316	118	434	2954.4	890.3	956.5	93.0	3.3

^1^Physical coverage as measured between the first and last marker on the chromosome (May 2006 assembly).

^2 ^Total length of the chromosome according to May 2006 assembly.

^3 ^SNP markers that have been assigned to chromosome 16, however, they do not show linkage to each other.

^4 ^LG1 represents linkage group E22C19W28E50C23, a linkage group that to date has not been assigned to a chromosome.

^5 ^GCT004 and rs16748775 denote markers that did not show linkage to any other marker in the dataset.

The map length in cM and the physical coverage in Mb are given for each chromosome.

Twenty out of the 138 microsatellites were excluded from the current map either because of low information content or because they showed inheritance errors that became apparent with the high-density marker map. Further, 35 SNPs were excluded, as they did not give reliable genotype calls, and additionally 33 SNPs out of the 384, were not considered in the current study as they are located on the Z chromosome. This updated sex-average autosomal linkage map now includes 434 genetic markers and spans totally 2954 cM, with an average distance between adjacent markers of 6.8 cM. The total map length is considerably shorter than expected from the old consensus map [18] but in good agreement with a new high-resolution, SNP-based consensus map for chicken [19]. It is clear that the inflated map length in the previous consensus map is because it was primarily based on microsatellite markers which have a higher rate of genotyping errors than Illumina Golden gate SNP assays as used in the present study and the new consensus map.

The current linkage map includes markers on chromosome 1–28, and in addition we also have markers on unassigned linkage group E22C19W28E50C23. Two genetic markers (GCT004, rs16748775) did not show linkage to any other marker in the dataset. It is possible that they are located on microchromosomes lacking other markers, or alternatively the genetic distance to neighboring markers was too far to be detected in the linkage analysis.

By calculating the distance between the first and last marker for each linkage group, we have physical coverage over 890.3 Mb corresponding to approximately 93% of the autosomal chicken genome assembly (May, 2006).

### Standard QTL analysis

Descriptive statistics for phenotypic traits analyzed in this study are presented in Table 1. Below we present results from standard genome-wide scans for marginal QTL effects on body-weight traits in the F_2 _population.

All QTL with significant marginal effect on body-weight traits are presented in Table 3. We found five QTL that were significant at the 5% genome-wide level for at least one body-weight trait and an additional two QTL that showed suggestive evidence for a QTL effect. The designation of these loci follows our previously described nomenclature (*Growth1-13*) given that they mapped to approximately the same genomic position [2].

**Table 3:** QTL with significant marginal effects on body-weight and growth traits.

QTL	Trait^1^	Chr:cM	F-value^2^	a ± SE^3^	d ± SE^3^	Var^4^
*Growth1*	GR56-70	1:442	18.1*	25.4 ± 4.2	1.8 ± 6.5	4.4
	BW70	1:445	11.6*	59.0 ± 12.2	5.0 ± 8.2	2.9
	GR42-56	1:450	6.4†	14.5 ± 4.2	5.0 ± 6.2	1.6
	BW56	1:452	7.3†	34.2 ± 9.2	7.8 ± 13.6	1.8
	GR0-14	1:469	7.0†	2.6 ± 0.8	-2.3 ± 1.3	1.6
	BW14	1:470	6.6†	2.4 ± 0.8	-2.7 ± 1.3	1.5
*Growth4*	BW14	3:61	9.1*	2.5 ± 0.7	-3.0 ± 1.1	2.1
	GR0-14	3:61	9.3*	2.4 ± 0.7	-3.1 ± 1.1	2.2
*Growth6*	GR42-56	4:31	9.0*	16.3 ± 3.9	-2.7 ± 5.9	2.2
	GR56-70	4:32	9.0*	15.9 ± 3.7	1.9 ± 5.7	2.3
	BW70	4:32	10.9*	51.4 ± 11.1	-7.2 ± 16.9	2.7
	BW56	4:33	9.8*	36.3 ± 8.3	-10.6 ± 12.6	2.4
	GR14-28	4:34	8.5†	8.3 ± 2.1	-3.7 ± 3.1	2.0
	BW28	4:34	8.2†	10.0 ± 2.5	-3.9 ± 3.8	1.9
	BW42	4:34	6.8†	18.3 ± 5.1	-7.1 ± 7.7	1.7
*Growth7*	GR28-42	4:86	6.8†	12.8 ± 3.5	-1.5 ± 5.1	1.7
*Growth9*	GR14-28	7:54	7.2†	6.1 ± 2.3	-9.0 ± 3.4	1.7
	BW28	7:55	7.8†	8.2 ± 2.7	-10.4 ± 4.1	1.8
	GR56-70	7:55	6.5†	12.2 ± 4.0	-10.9 ± 6.0	1.6
	BW42	7:76	9.2*	23.1 ± 5.5	-8.8 ± 8.0	2.3
	GR28-42	7:77	8.1†	13.1 ± 3.3	-4.7 ± 4.9	2.0
	BW56	7:78	11.9*	43.2 ± 9.0	-12.0 ± 13.0	2.9
	BW70	7:78	10.3*	53.6 ± 11.9	-11.6 ± 17.3	2.6
	GR42-56	7:81	9.3*	17.7 ± 4.1	0.2 ± 5.8	2.3
*Growth12*	BW28	20:46	6.5†	10.5 ± 2.9	1.3 ± 4.4	1.5
	BW42	20:54	7.0†	22.4 ± 6.1	-4.9 ± 9.8	1.7
	GR0-14	20:54	10.9*	3.8 ± 0.8	0.9 ± 1.3	2.5
	BW14	20:55	10.0*	3.7 ± 0.8	0.6 ± 1.3	2.3

^1^Growth rate (GR) measured as difference between body-weight measurements; body-weight (BW) was measured (in grams) at 14, 28, 42, 56 and at 70 days of age.

^2 ^* 5% genome-wide, † genome-wide suggestive significance level.

^3^The additive effect (a) represents half of the phenotypic difference between the two homozygous genotype classes (i.e. HH-LL) and the dominance effect (d) is defined as the average deviation of the heterozygotes (HL) from the phenotypic mean of the two homozygous genotypes.

^4^% of residual phenotypic variance explained by the QTL.

Three QTL regions influenced early growth (0–14 days) whereas four regions appeared to primarily affect later growth (14–70 days). For all QTL the allele originating from the HWS line increased growth. Each individual QTL explained only a small portion of the residual phenotypic variation in the F_2 _population, ranging from 1.5 – 4.4% (Table 3).

We detected a QTL influencing growth between 0 – 14 days and body-weight at 14 days of age with a QTL peak at 470 cM at chromosome 1. This QTL was not detected in our previous analysis and may represent a novel QTL. However, because it occurs in the vicinity of *Growth1 *we cannot exclude the possibility that it represents the same locus although *Growth1 *affects primarily later growth (Table 3).

**Figure 1:**
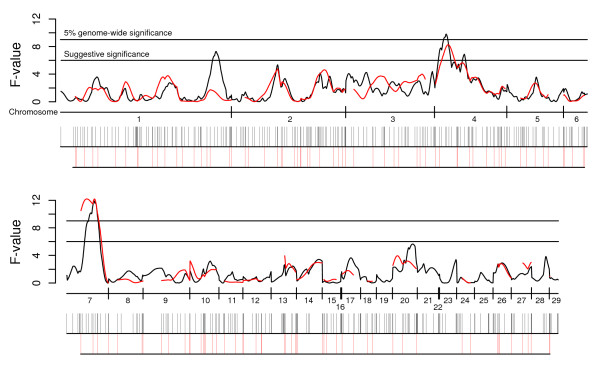
**Comparison of QTL results for body-weight at 56 days of age using the old microsatellite map and an improved map including 434 markers**. The black line represents F-values plotted for each cM throughout the genome for the new marker map while the red line displays F-values using the previous microsatellite-based map. The x-axis shows the start and end of each linkage group covered in the new map. Horizontal lines in the graph indicate the 5% genome-wide and the suggestive significance threshold. Black and red vertical bars underneath the x-axis indicate marker positions in the new and old map, respectively. Chromosome 29 denotes linkage group 'E22C19W28E50C23' that has not yet been placed on a chromosome.

Figure 1 visualizes the difference between the current QTL analysis and a QTL analysis performed for body-weight at 56 days of age using only microsatellite markers; the two maps have been aligned in order to provide a way to compare marker coverage and the results from the two QTL analyses. The overall agreement of the results from the two scans is excellent. We found a suggestive QTL on chromosome 1 (*Growth1 *interval) for body-weight at 56 days with the new map that was not detected with the old map due to the poor map coverage in the interval. No other chromosomal region lacking markers in the old map showed significant QTL effects. The localization of the major QTL on chromosome 7 is now much more precisely defined after we have added more markers on this chromosome (Figure 1).

### Detection of epistatic interactions

**Figure 2:**
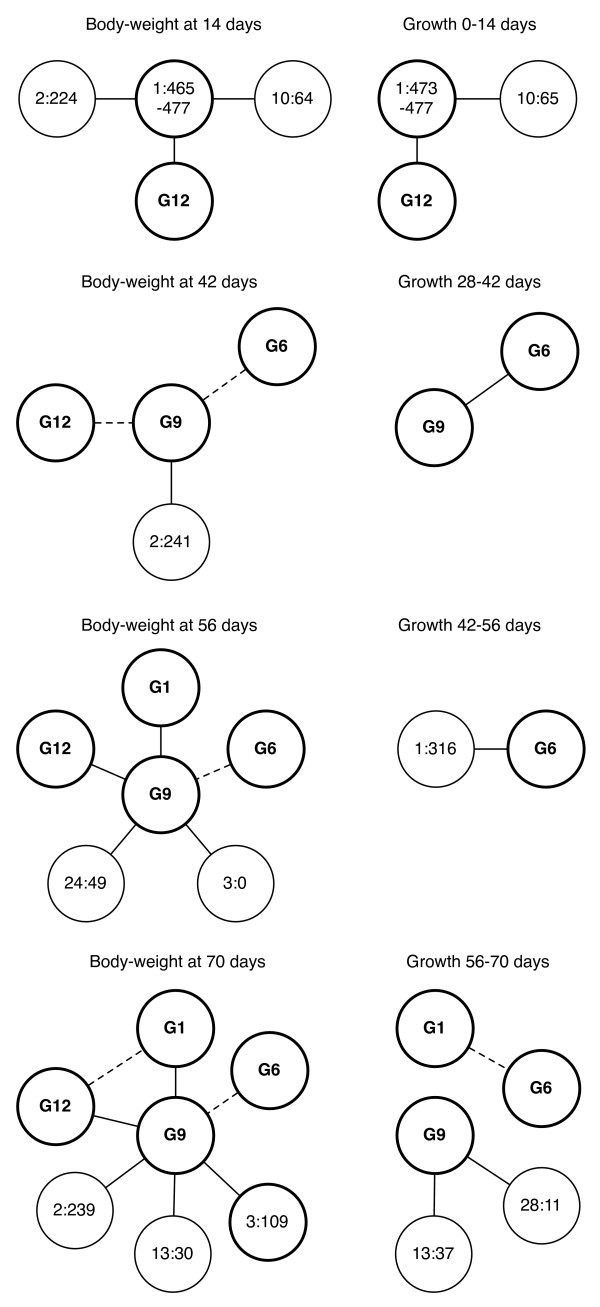
**Pairs of genome-wide significant interacting loci identified in the HWS/LWS intercross**. Bold circle line indicates that the locus had a significant (genome-wide or suggestive) marginal effect for at least one body-weight trait. Solid connection lines between loci (--) represent 1% significant interactions whereas dotted connection lines (--) indicate 5% significant interactions between loci.

**Figure 3:**
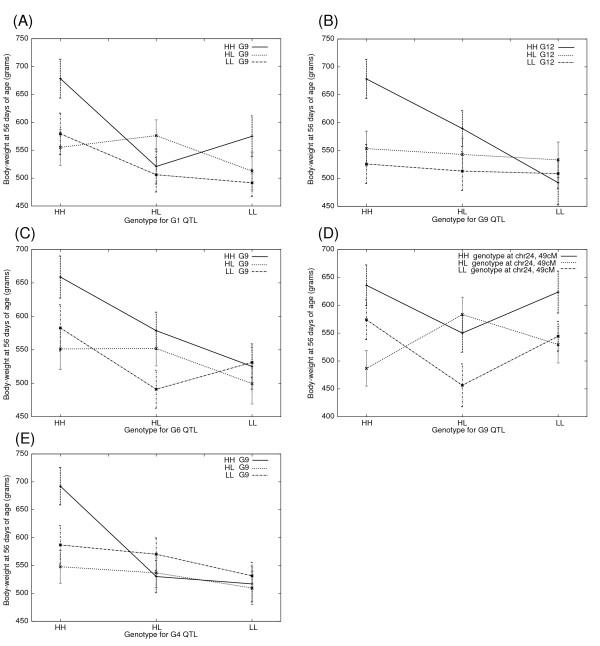
**Genotype-phenotype plots for genome-wide significant epistatic QTL pairs for body-weight at 56 days of age (A-E)**. The mean values of body-weight (in grams) for each of the nine possible allelic combinations are shown in the plot. The genotype classes for one QTL are listed on the X-axis (i.e. HH, HL and LL) and the curves represent each genotype class of the other QTL. Error bars represent the standard error of mean (s.e.m). HWS alleles are abbreviated as "H" and LWS alleles as "L".

We performed a genome-wide search for epistatic interactions, using the updated linkage map, by fitting a regression model including two-locus interactions [20]. An overview of all detected pair-wise interactions is presented in Figure 2. We analyzed ten growth traits (Table 1) and identified 19 QTL pairs where the interactions were significant at the 1% level and six additional pairs that were significant at the 5% level. The 25 detected pair-wise interactions, correspond to 15 unique pairs and seven pairs affected more than one growth trait. Nine of the 15 unique epistatic pairs involved interaction with the major QTL on chromosome 7 (*Growth9*). The genotype-phenotype map relationship for each pair was visualized by plotting mean values for each two-locus QTL genotype in a line graph (Figure 3; Additional File 2). As expected, the QTL pairs that affected multiple growth traits showed similar genotype-phenotype relationships across traits. There are some discrepancies between results from the current and previous epistatic analysis [4], when considering only body-weight at 56 days which was the age at selection. Originally, six genome-wide significant interacting loci were reported on chromosomes 1, 2, 3, 4, 7 and 20. The loci on chromosome 1, 4, 7 and 20 are still genome-wide significant in this analysis, though the loci detected on chromosome 2 and 3 are no longer significant using the new map. On the other hand, two new loci located in a previously uncovered part of chromosome 3 as well as a locus on chromosome 24 now reach significance above the threshold level. The genetic effects of the locus on chromosome 3 is, however, still highly dependent on the genetic background on chromosome 7, which makes us confident that this locus is still an important contributor to trait expression despite no longer reaching the stringent significance threshold in a genome-wide scan for epistasis.

**Figure 4:**
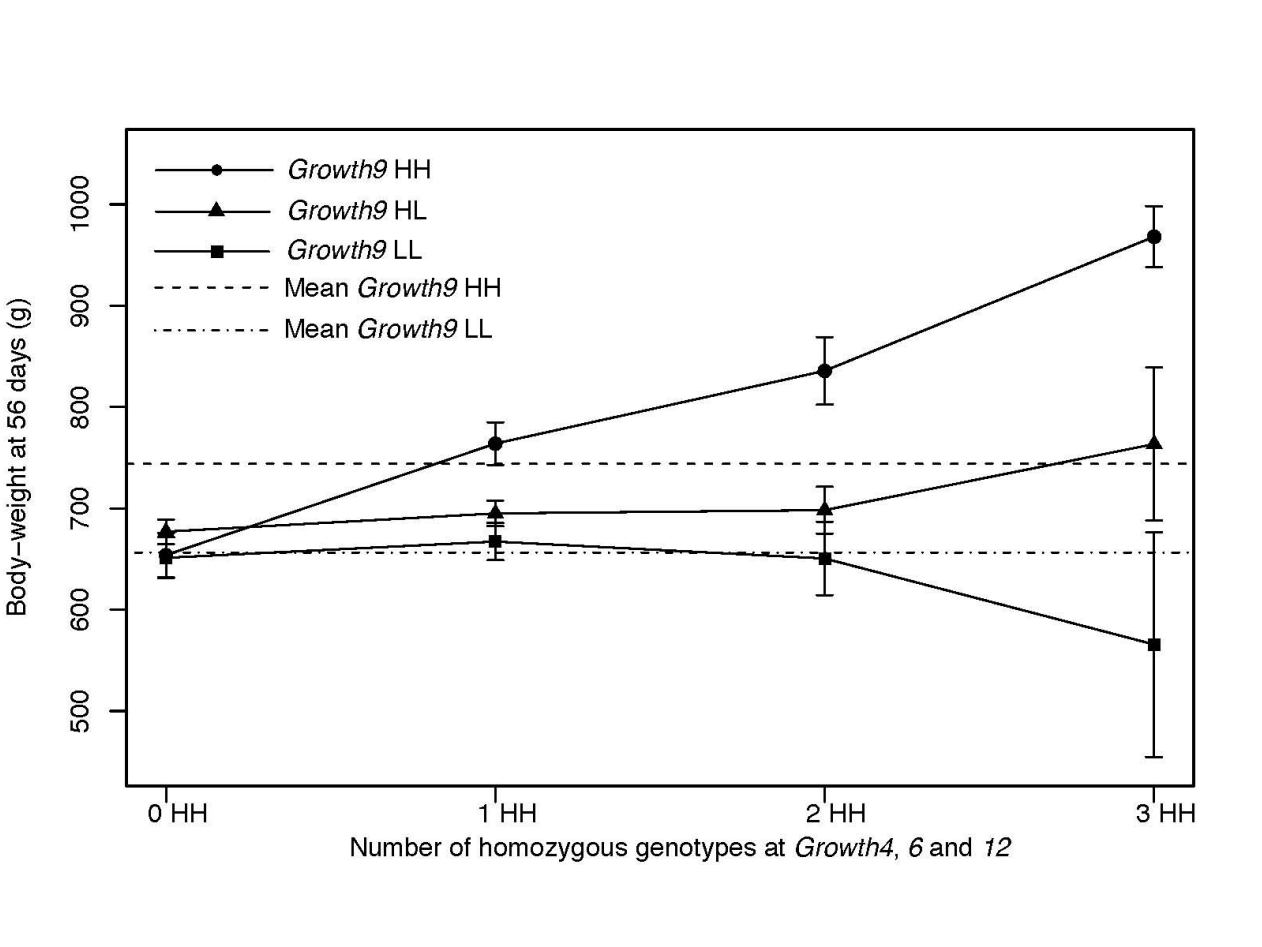
**A combined analysis of a four locus epistatic network affecting body-weight at 56 days of age**. Phenotypic mean values for body-weight at 56 days of age for alternative *Growth9 *genetic backgrounds plotted by the degree of HWS line homozygosity at *Growth4*, *Growth6 *and *Growth12*. Horizontal lines give the mean values for *Growth9 *HH and LL homozygotes. Error bars represent s.e.m. HWS line alleles are abbreviated as "H" and LWS alleles as "L".

In a combined analysis we studied the effect of *Growth4*, *Growth6 *and *Growth12 *conditional on the *Growth9 *background because it constitutes the major interaction network detected in this study (Figure 4). The results showed that increasing homozygosity for the HWS line allele (*H/H*) at *Growth4*, *Growth6 *and *Growth12 *had no phenotypic effect in a *Growth9 H/L *or *L/L *background. In contrast, chickens homozygous *H/H *at all three loci were 314 grams heavier than those homozygous *L/L *at all three loci, when the genetic background at the *Growth9 *locus was *H/H*. This demonstrates the strong epistatic interaction among these loci. An analysis including all five loci that interacted with *Growth9*, would have been desirable, however that would require a larger sample size than the ~800 F_2 _individuals we studied.

### Detection of selective sweeps using a dense SNP screen

We searched for potential selective sweeps by screening 15 birds from each line using a SNP panel comprising about 13,000 loci evenly distributed throughout the genome. Two birds from the LWS line were excluded from the analysis as results from a phylogenetic analysis indicated that there had been a sample mix up. We then calculated Fst and homozygosity values to identify the regions showing the most pronounced divergence between lines.

From the 13,000 SNP markers that were genotyped ~90% (11,275) provided reliable genotype calls (>95% call rate) and 42% of those (4,777 SNPs) showed allelic variation within or between the two lines. The average distance between polymorphic markers was 210 kb. A total of 1,917 SNPs segregated within both lines whereas 1,393 and 1,328 SNPs segregated only in the LWS or the HWS line respectively. Fixation of different alleles between lines was observed for 139 SNPs. The allele frequency changes observed show that considerable within-line fixation occurred during the course of the selection experiment. This is because both lines are derived from a common founder population in which all 4,777 SNPs must have been segregating. To test whether the distribution of the fixed SNPs deviates from a random distribution we computed the distances between consecutive markers in sets of 139 randomly chosen SNPs (10,000 times) and compared the random distribution to the observed one. A Kolmogorov-Smirnov test showed that the observed distribution deviates from the random distribution (P = 6 × 10^-13^). Hence, the distribution of fixed markers was clearly non-random across the genome as several clusters of fixed loci were detected. There were 36 clusters including two or more SNPs separated by less than 1 Mb between subsequent fixed SNPs and only 23 of the 139 SNPs were not closely linked to another fixed SNP. The QTL regions for body-weight at 56 days of age (Table 4) were two-fold enriched for SNPs as these regions constituted about 5% of the chicken genome (~50 Mb) but contained 10% of the fixed SNPs.

**Table 4:** Mean Fst and observed homozygosity across the genome and within QTL confidence intervals.

	Confidence interval				Homozygosity (Mean ± SD)	
			
Region	No. markers	Chr	Position (Mb)	Fst ± SD	HWS	LWS
Genome	4777	-	-	0.33 ± 0.29	0.74 ± 0.24	0.75 ± 0.22
All QTL	224	-	-	0.38 ± 0.33	0.75 ± 0.24	0.81 ± 0.22
*Growth1*	17	1	171.9 – 176.0	0.51 ± 0.38	0.76 ± 0.21	0.98 ± 0.05
*Growth6_7*^a^	93	4	5.1 – 35.0	0.40 ± 0.35	0.78 ± 0.24	0.82 ± 0.21
*Growth9*	47	7	21.8 – 33.3	0.45 ± 0.29	0.79 ± 0.24	0.71 ± 0.28
*Growth12*	67	20	6.0 – 12.0	0.29 ± 0.30	0.67 ± 0.23	0.86 ± 0.16

^a ^*Growth6 *and *Growth7 *were combined in the analysis as their confidence intervals based on the one-LOD drop method overlapped.

Only intervals with significant QTL for later growth (14 – 70 days) were considered.

We calculated Fst-values for each marker located in QTL intervals for body-weight at 56 days of age and compared the mean Fst to the average genome-wide Fst-values. The QTL regions were here defined by their confidence intervals calculated by the one-LOD drop method [17]. The results are presented in Table 4 together with observed homozygosity estimates. Only minor differences were found between the average Fst-values in QTL intervals and the genome average. This suggests that the size of the haplotype blocks associated with a causative QTL mutation are small compared to the size of the QTL confidence intervals obtained in our F_2 _segregation analysis. However, the confidence interval for *Growth1 *on chromosome 1 showed a markedly higher divergence between lines and near homozygosity within the LWS line (Table 4). This result suggests that a selective sweep has occurred in the LWS line. The interval showing near fixation within the LWS line is 3.5 Mb in size and spans from 173.4 Mb to 176.9 Mb.

## Discussion

The results of the present study are consistent with the major conclusions obtained in our previous QTL analysis of this large intercross pedigree based on a less complete linkage map [2-4]. Despite the large phenotypic differences between the High-Weight and Low-Weight selected lines we were not able to detect any major QTL with a large marginal effect. Only five loci, *Growth1*, *4*, *6*, *9 *and *10*, reached genome-wide significance for at least one body-weight or growth trait and none explained more than 4.4% of the residual variance for a growth-related trait (Table 3). However, the importance of epistatic interaction as a mechanism contributing significantly to the remarkable response to selection was further strengthened because additional pairs of interacting loci were detected in the present study. We are currently using an Advance Intercross Line (AIL) to replicate and further explore interactions among these loci.

The HWS and LWS lines originated from the same base population that was formed by crossing seven partially inbred lines of White Plymouth Rock broiler chickens [1]. That the results of the QTL analysis infer that no QTL with large individual effects segregated in the base population is consistent with the steady selection response in both directions that have been obtained during the 40 generations of divergent selection [1,2]. This pattern is also consistent with a rather slow change in allele frequencies at QTL during the course of selection or a gradual release of selectable additive genetic variation from the epistatic loci [21].

The remarkable response to the divergent selection for juvenile body-weight in the HWS and LWS lines must reflect allele frequency changes between lines at those loci that have responded to selection. The chromosomal regions harboring such loci are expected to show reduced variation within lines and also a higher divergence between lines. Such selective sweeps are caused by hitchhiking of closely linked loci during selection [22]. The size of a selective sweep depends on the number of generations that have passed since the QTL mutation occurred and the local recombination rate in the region. Thus, a recently derived mutation will be associated with a large haplotype block whereas an old mutation that well predates the initiation of the selection experiment is expected to be associated with a smaller haplotype block.

The outcome of the analysis of Fst and homozygosities based on the screening of 13,000 SNPs in the HWS and LWS lines are consistent with the results of the QTL analysis and the observed selection response. We found only minor differences in the average homozygosity as well as average Fst values within QTL intervals compared with the genome average (Table 4). The result suggests that selection has primarily been acting on standing genetic variation that existed well before the initiation of the selection experiment, which is supported by results of a recent simulation study exploring the potential role of epistatic interactions in response to directional selection [21]. The causal mutation will occur in many haplotype combinations if it has been transmitted through a large number of meiotic events before it reaches a high allele frequency in the selected population and as a consequence the selective sweep will be short and not detected by an Fst analysis based on the rather sparse marker set used in this study (~1 polymorphic SNP/200 kb). The results suggest that the release of genetic variance due to epistatic interaction [4] is a more likely explanation for the long-term selection response in these lines rather than the occurrence of new mutations during the course of selection [23].

An indication of a selective sweep was, however, observed for *Growth1 *on chromosome 1 where there was a higher Fst value between lines than the genome average. The confidence interval for *Growth1 *was smaller than for the other growth QTL which facilitated the detection of footprints of selection. The homozygosity in this QTL interval for the HWS line was close to the genome average whereas the region showed almost complete fixation in the LWS line. The pattern suggests that while this locus has contributed to the selection for low growth in the LWS line it may have been selectively neutral in HWS line. The causal mutation(s) for this QTL is expected to be located within this region of high homozygosity. The region with near homozygosity in the LWS line spans from position 173.4 to 176.9 Mb on chicken chromosome 1, a region still too large and containing too many genes to pin-point strong positional candidate genes.

To be an exhaustive search for QTLs, a genome scan should be based on a marker set that covers the entire genome. The map used in the current study covered 93% of the assembled chicken genome a clear improvement to the ~80% coverage achieved with our previous microsatellite-based map. In particular, we have added markers on six microchromosomes that previously lacked markers. There are, however, still seven microchromosomes lacking markers in the current map. This is because they are missing in the genome assembly and no markers from these chromosomes have yet been reported. These missing microchromosomes are all small, on the order of five Mb or less but are expected to have a higher gene density than the macrochromosomes [24]. It is apparent that parts of the chicken genome, including some microchromosomes, are difficult to clone in bacterial vectors [24,25] and this is the reason why they are missing from the current genome assembly. Hopefully, the use of new sequencing technologies that do not require vector-based cloning will allow us to fill the holes in the current chicken genome assembly to eventually making a complete genome scan feasible.

If we compare our current QTL analysis based on an improved linkage map with our previous QTL analysis [2] there are as many as six previously reported loci (*Growth2, Growth3, Growth8, Growth10, Growth11 *and *Growth13*) that did not reach statistical significance in the present study. Our previous analysis included family as a fixed effect in the regression model whereas we decided to not include family as a fixed effect in the statistical model used in the present study. To test whether the new information provided by the improved linkage map or the change in regression model caused this discrepancy we reran the analysis including family as a fixed effect for those growth loci that we could not replicate. With a QTL model including Family as a fixed effect, the F-value associated with *Growth2 *increased from 5.4 to 6.3 and therefore reached the suggestive significance threshold. For *Growth3*, the F-value increased from 4.5 to 6.0 with family effect in the model. A more dramatic increase in significance was observed for *Growth8 *since the F-value increased from not reaching even the suggestive threshold to become genome-wide significant with an F-value of 9.3 when the Family effect was included in the model. It is very likely that these three loci (*Growth2, 3 *and *8*) represents true QTL effects and at each locus the allele from the HWS line is increasing growth as expected from the line differences in growth. In contrast, for *Growth10*, *11 *and *13 *there was only a minor difference in statistical significance between the two models and in these cases it is more likely that the previous suggestive evidence for QTL at these positions in fact were false positives.

## Conclusion

The eight-fold difference in body-weight at selection age between two divergently selected chicken lines is determined by many QTL each with a small individual effect. Although no major QTL explaining a large proportion of the residual phenotypic variance was found, a network of interacting loci with a large effect was identified using the current extended linkage map. Furthermore, results from the QTL study was consistent with our analysis of Fst and homozygosity using a marker set including ~13,000 SNP markers. The results from that analysis failed to detect any major selective sweeps within QTL region suggesting that selection has been acting on standing genetic variation present in the founder population rather than recently derived mutations.

## Authors' contributions

PW organized the SNP genotyping of the pedigree, constructed the linkage map, contributed to the QTL analysis and the SNP analysis, summarized the results and drafted the manuscript. ÖC supervised and contributed to the QTL analysis. XT performed statistical analysis of SNP data, assisted with the organization of the genotype data. A-CS supervised the genotyping of the pedigree at Uppsala University. MF, ML and IG generated the SNP data used to detect selective sweeps. PBS designed the study together with LA, organized and produced the intercross and supervised the collection of all phenotypic measurements. LA supervised the molecular and the statistical analysis performed in Uppsala, revised and made final adjustments of the manuscript. All authors read and approved the final manuscript.

## Supplementary Material

Additional file 1 **Genetic markers included in the new linkage map**. Positions of markers are shown in Kosambi cM and in Mb according to the current genome assembly (May, 2006). Click here for file

Additional file 2 **Genotype-phenotype interaction maps for all epistatic QTL detected in the intercross for growth traits**. Mean values of body-weight (in grams) for each of nine possible allelic combinations are shown in the plots. The genotype classes for one QTL is given on the X-axis (i.e. HH, HL and LL) and the curves represent the genotype classes of the other QTL. Error bars represent the standard error of mean (s.e.m.). HWS alleles are abbreviated as "H" and LWS alleles as "L". The figure is arranged so that all traits significant for the same unique interaction pair are displayed together. **(A) **QTL at Chr1, 316 cM – G6, **(B) **QTL at Chr1, 565 cM – Chr2, 224 cM, **(C-D) **Chr1, 473 cM – Chr10, 64 cM, **(E-F) **G12 – Chr1, 477 cM, **(G) **G9 – Chr24, 49 cM, **(H) **G9 – Chr28, 11 cM, **(I-K) **G9 – G12, **(L) **G1 – G12, **(M-P) **G9-G6, **(Q – R) **G9 – Chr2, 240 cM, **(S-T) **G9 – G1, **(U-V) **G9 – Chr3, 0 cM, **(W-X) **G9 – chr13, 30 cM, **(Y) **G1 – G6. Click here for file
