# Supplementary material for: Genetic analysis of an F2 intercross between two chicken lines divergently selected for body-weight
Source: BMC Genomics. 2009 May 27;10:248. doi: 10.1186/1471-2164-10-248 (PMC2695486; doi:10.1186/1471-2164-10-248)
Supplement: Additional file 1 — Genetic markers included in the new linkage map. Positions of markers are shown in Kosambi cM and in Mb according to the current genome assembly (May, 2006). [file 1471-2164-10-248-S1.pdf]

Marker	Chromosome[a]	cM	Mb[b]
RBL438	1	0.0	0.3
rs14080307	1	36.0	0.7
rs13824562	1	41.7	2.4
rs13621730	1	47.5	6.1
rs14795085	1	57.1	13.8
rs14792587	1	63.4	15.9
RBL1258	1	68.6	17.2
rs13837741	1	78.1	24.6
rs13842499	1	89.9	29.1
MCW0254	1	92.3	31.4
rs13847756	1	101.1	34.9
rs13851434	1	115.4	38.0
rs14816632	1	127.1	45.1
rs14818658	1	135.1	47.8
rs13865452	1	149.3	52.5
LEI0146	1	159.7	55.3
rs15276085	1	167.5	57.9
rs14828994	1	170.1	58.6
rs14832321	1	176.3	61.8
MCW0018	1	187.9	65.7
rs13880460	1	196.0	68.6
rs13883708	1	210.5	71.5
rs13819552	1	218.2	74.0
rs13819560	1	218.6	74.0
RBL2228	1	219.0	74.2
RBL4547	1	219.2	74.3
rs13834451	1	220.3	9.9
rs13997062	1	221.3	75.0
MCW0058	1	222.9	79.0
LEI0071	1	223.7	79.5
rs13887893	1	230.5	80.9
rs14693408	1	230.8	81.4
rs15318252	1	235.1	82.3
rs13583965	1	250.6	86.1
rs13646704	1	255.3	87.6
ADL0367	1	266.7	91.6
rs15340573	1	268.1	92.1
BU-1-1-85821084	1	273.9	na
LEI0108	1	280.6	97.1
rs15353627	1	282.9	99.6
rs14864919	1	286.9	104.2
rs13914871	1	291.5	107.8
rs13918284	1	301.1	110.5
MCW0200	1	302.4	111.3
rs13921245	1	310.4	113.5
rs14876047	1	323.6	116.9
rs15402046	1	333.8	122.8
ADL0353	1	337.6	124.2
rs14884222	1	351.5	128.7

RBL3441	1	352.7	129.1
MCW0036	1	353.5	129.3
RBL1448	1	354.3	130.8
rs14887388	1	360.4	134.4
rs13625427	1	372.0	138.0
LEI0107	1	387.4	143.7
rs13951254	1	391.0	147.8
rs13954000	1	401.1	150.1
rs13958072	1	407.0	156.9
rs14905876	1	407.7	157.1
LEI0079	1	410.6	158.4
LEI0162	1	426.3	169.1
ADL0245	1	434.6	171.7
rs13552040	1	435.3	171.9
rs15502284	1	448.0	175.2
rs14920831	1	455.4	177.7
rs15516954	1	466.8	181.1
rs13982336	1	473.1	182.7
IAP1-1-174542266	1	481.1	na
rs14931269	1	483.5	189.3
LEI0134	1	490.5	191.8
rs14935422	1	493.2	193.3
RBL2401	1	494.6	195.1
GCT0001	1	497.1	197.6
rs15054320	2	0.0	2.0
rs13534858	2	14.8	4.4
rs14134143	2	25.7	7.0
rs15884716	2	30.0	8.8
rs14139143	2	35.2	11.6
ADL0190	2	39.0	15.3
rs14143285	2	39.6	15.7
MCW0247	2	47.6	19.4
rs15910200	2	48.0	19.4
rs15075023	2	60.3	24.1
rs15077078	2	67.2	26.9
rs15081191	2	81.5	32.4
rs15083693	2	90.0	35.0
MCW0063	2	95.2	37.9
rs13670674	2	103.1	41.2
rs13671945	2	110.7	43.4
rs14178920	2	127.5	47.9
rs15099222	2	132.4	50.4
MCW0062	2	134.1	56.1
MCW0293	2	143.4	59.6
rs15105085	2	146.6	60.5
rs15107772	2	155.2	63.4
rs14196021	2	160.4	65.5
rs14200463	2	177.1	68.9
LEI0096	2	183.2	71.0
rs16039935	2	186.2	77.5

ADL0157	2	191.7	80.7
MCW0179	2	199.9	86.6
MCW0087	2	201.7	87.2
rs15126683	2	207.7	91.2
rs14219471	2	216.4	93.7
UMA02	2	220.1	96.1
RBL4114	2	224.4	100.0
LEI0147	2	228.2	101.6
RBL3577	2	229.7	102.2
RBL3422	2	229.9	102.2
rs14225365	2	231.4	103.3
rs16092012	2	242.8	108.0
RBL8	2	251.0	111.1
rs14232457	2	253.2	112.1
RBL2593	2	253.9	112.8
rs13796585	2	257.1	114.7
MCW0234	2	257.3	114.9
rs16107541	2	263.0	117.4
rs16108913	2	265.5	119.0
rs14240558	2	273.3	122.4
rs14242430	2	276.2	125.0
rs13731505	2	286.4	129.5
MCW0245	2	296.2	131.9
RBL3465	2	302.2	na
rs16136501	2	307.3	135.5
LEI0070	2	311.4	139.6
rs13762257	2	319.6	143.8
MCW0320	2	323.2	145.8
rs14260168	2	327.4	149.7
LEI0031	2	330.8	153.3
MCW0311	2	331.7	153.7
rs13758611	2	332.6	154.4
rs14308203	3	0.0	2.4
rs14310908	3	14.9	4.6
MCW0169	3	23.0	6.6
rs16222521	3	37.3	9.5
rs14084016	3	50.5	15.0
rs13717180	3	60.7	18.8
rs14326182	3	77.5	24.0
MCW0222	3	78.5	24.2
rs14330022	3	86.0	27.8
rs14336675	3	105.1	33.7
rs15321683	3	109.1	35.5
ADL0155	3	112.8	37.6
rs14341292	3	113.1	37.8
rs13675715	3	127.0	42.4
ADL0371	3	140.0	45.2
MCW0004	3	154.2	51.6
rs16281414	3	163.7	57.1
RBL2668	3	172.3	60.2

rs15366849	3	172.9	61.3
rs14367728	3	178.6	65.6
rs15375524	3	182.2	68.3
rs14376988	3	200.9	75.5
MCW0224	3	207.2	79.8
ADL0024	3	216.5	83.4
rs13696669	3	224.1	87.9
rs14391415	3	227.6	90.5
MCW0207	3	231.1	92.9
rs14397663	3	238.7	96.8
rs14400934	3	248.6	100.1
LEI0065	3	255.2	103.8
rs13702405	3	256.6	103.9
rs14408135	3	262.8	107.3
rs15471980	4	0.0	1.0
ADL0143	4	1.5	1.4
ADL0317	4	14.3	3.5
rs14421644	4	20.3	4.1
rs15484517	4	30.7	6.8
rs13643096	4	33.7	9.4
rs14428592	4	40.8	11.4
rs15500313	4	49.0	13.5
rs14435605	4	64.2	17.7
ADL0145	4	65.5	17.9
MCW0251	4	67.0	19.2
rs14438666	4	68.4	24.6
rs15522441	4	74.2	29.5
rs14446940	4	86.3	33.1
rs15542429	4	97.4	37.0
rs15544037	4	98.0	38.1
MCW0091	4	102.5	39.7
rs15549573	4	106.1	40.9
rs15554707	4	110.6	44.0
LEI0125	4	111.4	44.7
rs14462677	4	119.7	47.8
rs15581865	4	140.3	55.0
LEI0076	4	151.4	62.1
rs14479812	4	153.4	63.2
rs15607084	4	161.9	67.9
RBL1352	4	172.7	71.6
rs14488074	4	173.0	71.6
LEI0148	4	173.7	na
rs14491521	4	182.4	79.5
rs13664708	4	185.3	81.1
MCW0098	4	186.8	81.5
rs13666021	4	192.3	84.6
rs16444662	4	193.8	85.5
LEI0085	4	195.3	85.9
LEI0073	4	204.5	88.4
rs15643977	4	210.8	91.7

rs14508809	5	0.0	1.6
rs15343523	5	6.5	5.5
rs14511732	5	19.9	9.4
rs15661711	5	28.1	12.1
rs13757471	5	33.1	14.4
MCW0193	5	39.1	15.9
RBL1369	5	43.2	17.3
RBL2307	5	43.8	17.7
rs15674552	5	48.0	18.5
rs14693539	5	50.8	19.6
MCW0038	5	55.6	21.6
rs15679760	5	61.8	24.4
rs13584119	5	75.4	29.8
MCW0078	5	81.2	31.9
rs13585490	5	84.7	36.3
rs13586869	5	102.3	41.0
MCW0029	5	106.0	44.2
rs16498806	5	106.0	44.7
LEI0149	5	108.7	46.5
MCW0081	5	120.5	51.4
rs16514663	5	136.3	56.1
rs15743161	5	151.1	59.1
rs16522750	5	164.1	61.3
LEI0192	6	0.0	4.0
MCW0118	6	0.7	4.0
rs15758001	6	6.7	5.0
rs16537104	6	16.5	8.5
rs15778112	6	21.1	11.7
rs15780532	6	23.2	12.9
MCW0250	6	24.7	16.6
rs15800133	6	44.5	25.4
rs14588347	6	52.5	28.6
ADL0377	6	56.2	29.7
rs14591306	6	60.3	31.3
LEI0196	6	60.9	31.3
rs14593897	6	69.7	33.6
rs14598438	7	0.0	1.5
rs13739720	7	16.1	3.6
rs13740451	7	31.0	5.4
rs15837786	7	35.6	9.0
rs13783972	7	36.6	11.3
MCW0120	7	40.0	11.9
rs14608276	7	44.5	13.1
rs15852323	7	54.7	17.2
rs15856536	7	61.5	20.6
rs14616506	7	71.7	24.6
ADL0279	7	74.3	25.3
rs15870814	7	80.4	28.0
MCW0236	7	87.6	29.7
rs16604163	7	88.0	29.8

rs15882678	7	97.8	33.3
rs16612776	7	111.8	35.8
ADL0169	7	120.2	38.0
rs16620523	8	0.0	3.8
MCW0305	8	25.7	7.0
rs14642627	8	36.0	14.6
rs14650356	8	71.3	22.3
rs14652607	8	79.9	24.3
rs16644389	8	83.1	25.9
rs14655901	8	90.1	27.4
ADL0172	8	97.2	28.8
rs15940094	8	98.7	29.2
ADL0278	8	100.3	29.9
rs16650965	9	0.0	1.7
MCW0024	9	53.2	4.9
rs14669284	9	59.0	5.4
rs15957507	9	65.9	8.3
rs14662996	9	69.5	10.6
MCW0135	9	73.0	13.7
rs14674285	9	85.6	15.1
rs14676340	9	100.3	16.9
rs14678700	9	107.9	20.6
ADL0219	9	121.4	22.6
MCW0134	9	135.8	24.8
MCW0228	10	0.0	0.7
rs13999825	10	15.0	1.6
ADL0209	10	31.7	4.1
MCW0194	10	38.8	6.7
rs14003423	10	40.0	7.4
rs13799452	10	41.2	9.6
MCW0067	10	41.9	9.6
rs14006050	10	46.2	12.6
ADL0272	10	46.5	12.7
rs14009067	10	59.0	15.2
ADL0106	10	65.0	16.5
rs15589031	10	75.0	18.9
ADL0158	10	77.1	19.4
rs14013921	10	84.3	21.4
rs14957451	11	0.0	0.9
rs14958678	11	11.5	1.9
rs14019836	11	14.1	5.2
ADL0123	11	15.2	5.7
rs14022357	11	27.6	na
rs14963832	11	33.9	10.9
rs14025658	11	38.1	13.8
RBL3085	11	45.5	16.9
ADL0308	11	48.7	17.4
rs14966910	11	52.9	18.7
ABR0037	11	69.5	20.6
rs14030286	12	0.0	0.7

ADL0372	12	0.2	0.7
rs13621493	12	26.7	2.5
LEI0131	12	37.5	4.9
rs14035356	12	38.0	5.8
rs14039242	12	44.0	9.2
ADL0044	12	54.1	12.3
LEI0099	12	54.5	12.4
rs15662367	12	63.4	14.7
rs14046447	12	76.1	17.3
rs14048107	12	81.6	18.7
rs15675703	13	0.0	0.7
rs14990028	13	30.2	5.2
rs14051703	13	32.0	6.9
ADL0147	13	34.0	8.1
rs14994099	13	35.0	8.3
RBL3916	13	36.8	8.9
ROS00325	13	38.9	9.4
MCW0213	13	42.0	10.4
MCW0315	13	59.0	14.4
rs14998975	13	60.9	14.9
rs14064615	13	70.6	17.0
ADL0225	13	71.7	17.2
rs15000764	13	73.9	18.4
ADL0118	14	0.0	0.1
rs13530010	14	0.1	0.3
MCW0296	14	0.9	1.5
rs13531951	14	28.3	5.1
rs15008964	14	34.4	6.8
rs15010436	14	37.1	8.9
LEI0066	14	74.5	14.7
rs15763765	15	0.0	1.9
LEI0083	15	3.9	2.8
rs15019494	15	5.7	4.0
ADL0039	15	11.6	5.7
rs15021350	15	15.0	6.4
rs14092594	15	25.4	8.2
rs15024160	15	31.0	9.7
MCW0211	15	40.0	10.8
rs15026117	15	53.3	11.9
rs15788101	16	-	0.1
rs15026773	16	0.0	0.1
rs15026791	16	0.0	0.1
ADL0199	17	0.0	0.7
rs14104304	17	4.4	1.7
rs13734134	17	19.9	3.5
ADL0149	17	33.9	5.7
rs15031008	17	35.8	5.9
rs15029104	17	48.5	8.1
rs15027463	17	54.6	9.6
rs15027195	17	54.7	10.1

rs14106100	18	0.0	1.0
rs14108386	18	15.1	2.7
MCW0217	18	18.8	3.2
rs13507726	18	32.3	4.9
ADL0184	18	32.9	5.0
rs14113801	18	41.3	8.0
rs15833225	18	45.1	9.0
rs14115711	19	0.0	0.9
rs14116385	19	7.3	1.5
rs14118674	19	22.8	3.3
rs15047231	19	27.7	4.3
rs15048343	19	34.4	5.6
rs15848432	19	37.3	6.1
rs14122543	19	40.6	7.2
rs14124079	19	46.6	9.6
MCW0119	20	0.0	0.4
rs15169246	20	7.3	0.9
ADL0125	20	28.4	3.1
rs15171181	20	32.4	3.4
rs14272231	20	37.9	4.7
rs15173767	20	43.8	6.2
rs14274909	20	45.3	7.1
rs14276041	20	48.3	8.1
rs14278769	20	65.0	10.8
rs13635174	20	68.6	11.9
MC3R-NA-1035	20	68.8	12.0
MC3R_PYRO	20	68.8	12.0
rs13795178	20	71.8	13.9
rs13602346	21	0.0	0.4
rs14282175	21	14.0	1.5
rs14283139	21	18.2	2.6
rs15182893	21	25.0	3.4
rs16181928	21	46.1	5.2
rs14286166	21	61.5	6.6
rs15191004	22	0.0	2.9
rs13820393	22	2.7	3.4
rs14287506	23	0.0	0.2
rs15194003	23	7.9	1.0
rs14289029	23	16.8	1.8
rs15205573	23	47.4	5.0
rs15207546	23	49.7	5.6
rs13603923	24	0.0	0.5
rs16194400	24	3.1	1.8
RBL1526	24	7.4	2.3
rs14294624	24	11.4	2.9
ROS00302	24	15.4	na
LEI0069	24	39.9	5.1
rs14297486	24	51.5	6.1
rs16740409	25	0.0	na
RBL3432	25	17.4	0.8

RBL4442	25	38.6	na
RBL625	25	40.4	na
rs14710054	25	40.5	na
RBL2937	25	53.1	1.9
rs14416247	26	0.0	0.4
SNP-152-100-21801-S-1	26	2.0	na
rs16200368	26	6.3	1.4
MCW0286	26	13.6	1.9
MCW0209	26	15.9	2.1
MCW0069	26	18.9	2.4
rs16202845	26	32.4	3.3
LEI0074	26	52.9	4.7
RBL280	27	0.0	0.4
rs15241802	27	0.9	1.2
rs14301476	27	4.7	1.5
rs13620274	27	9.0	2.1
MCW0076	27	33.0	na
rs14303776	27	38.6	3.4
ADL0376	27	47.6	3.9
MCW0292	27	57.4	na
rs16208538	27	59.0	4.2
rs15245588	28	0.0	0.7
rs14306112	28	33.7	2.1
rs16211629	28	37.7	2.4
MCW0227	28	41.5	2.5
rs16212707	28	51.7	4.1
MCW0188	LG1	0.0	na
ROS00306	LG1	1.9	na
RBL3889	LG1	3.3	31.4
RBL4985	LG1	5.1	38.3
RBL4854	LG1	22.9	42.0
rs15989709	LG1	23.5	na
RBL1494	LG1	25.5	0.9
GCT004	Chr_UN	-	na
rs16748775	Chr_UN	-	na

[a] Chr_UN corresponds to sequence contigs that have not been incorporated in the genome assembly. LG1 represents linkage group E22C19W28E50C23. No linkage was detected between rs15788101 and the two other markers located on chr16.

[b] NA indicates that there is no position on the current assembly (May 2006).
