# Supplementary figures and images for: Genetic analysis of an F2 intercross between two chicken lines divergently selected for body-weight
Source: BMC Genomics. 2009 May 27;10:248. doi: 10.1186/1471-2164-10-248 (PMC2695486; doi:10.1186/1471-2164-10-248)

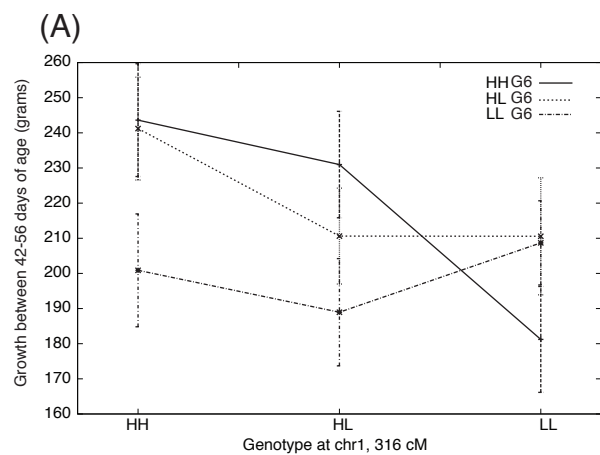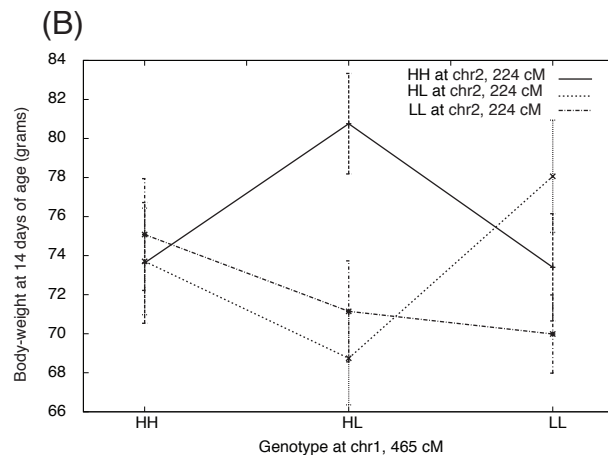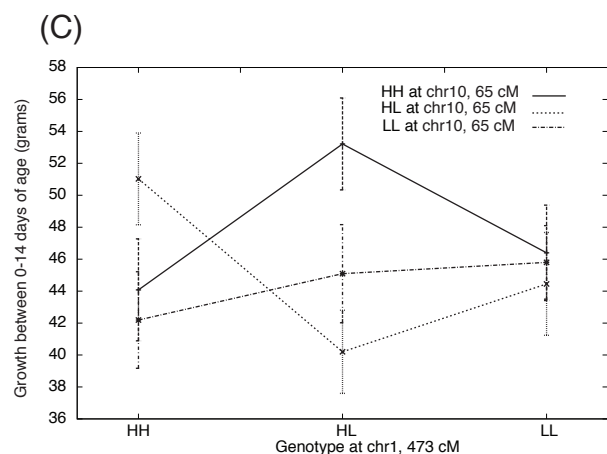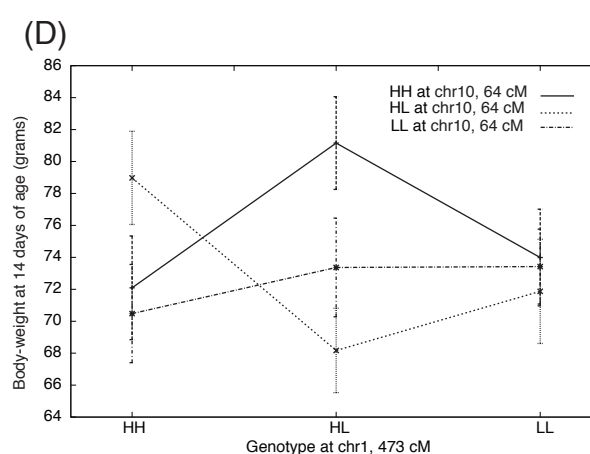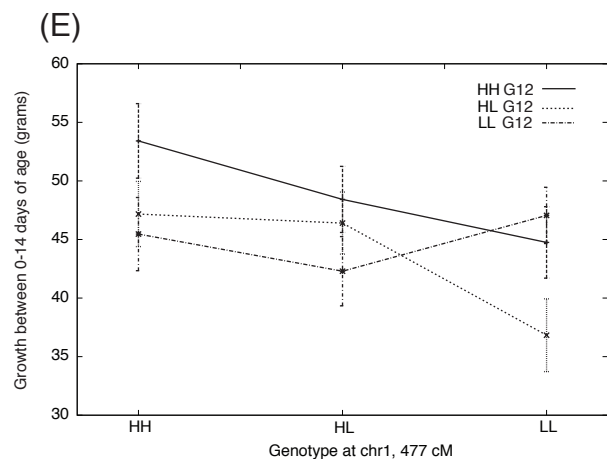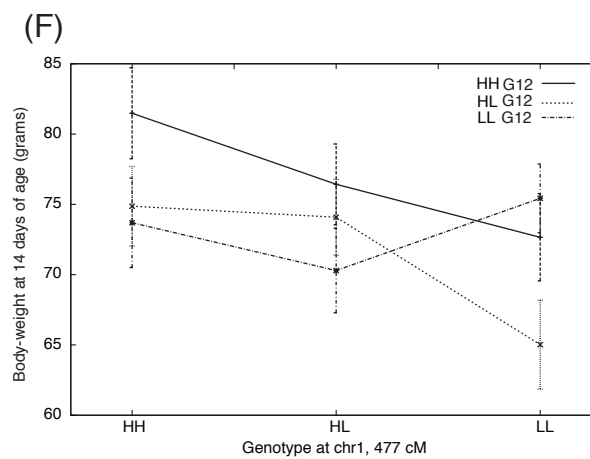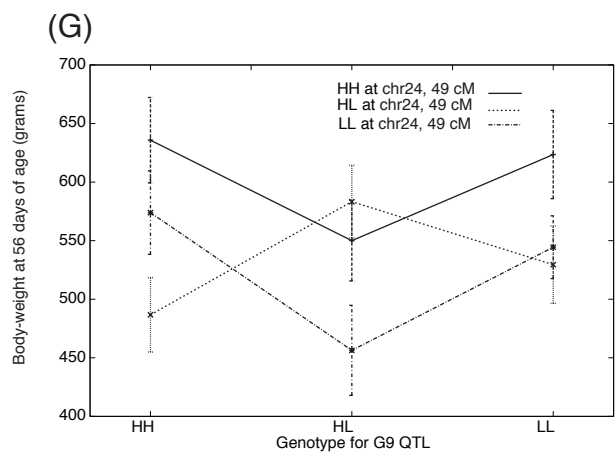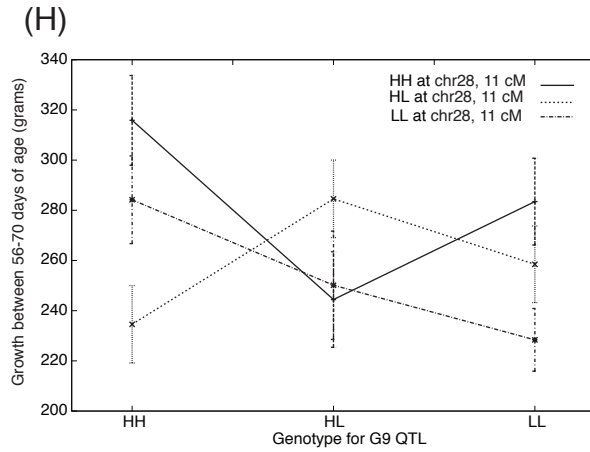

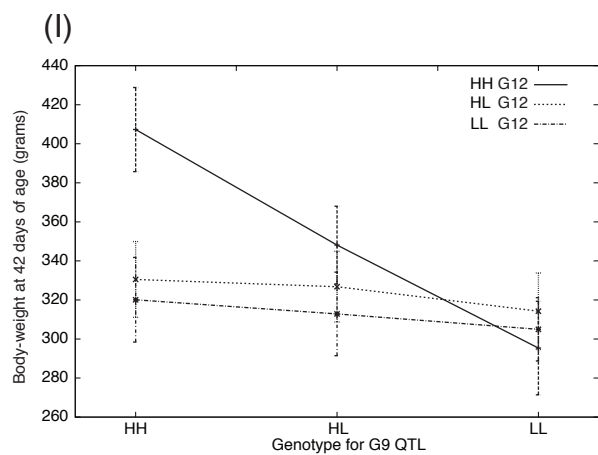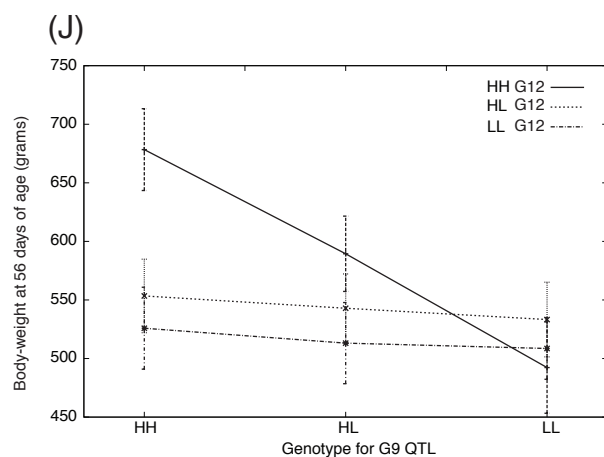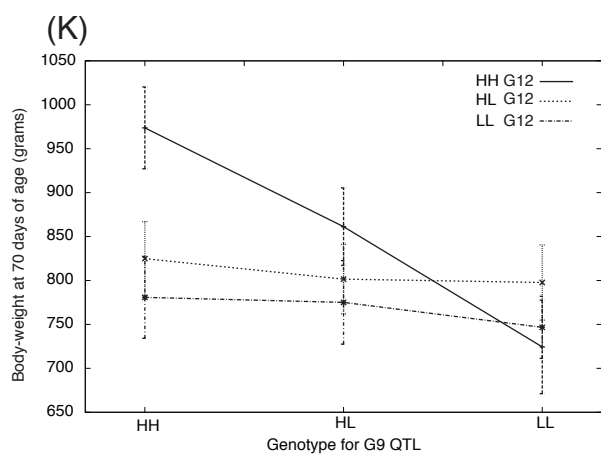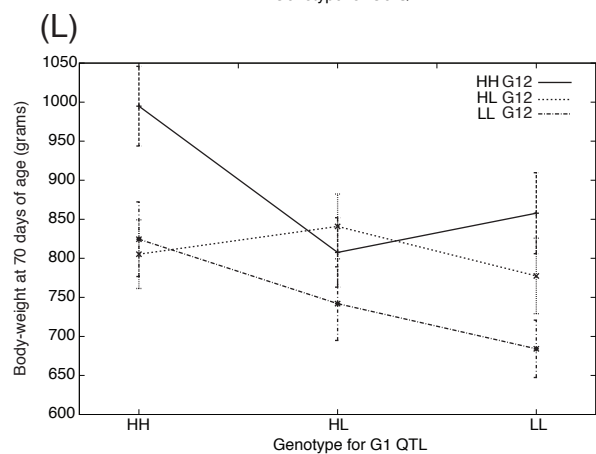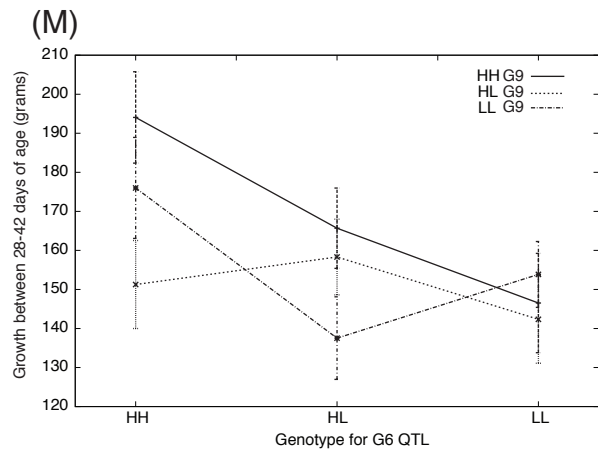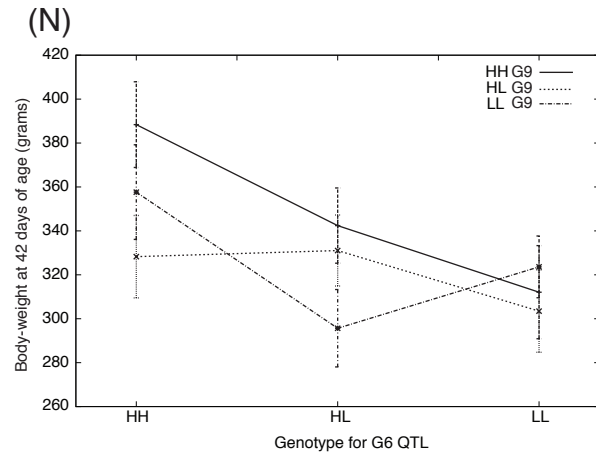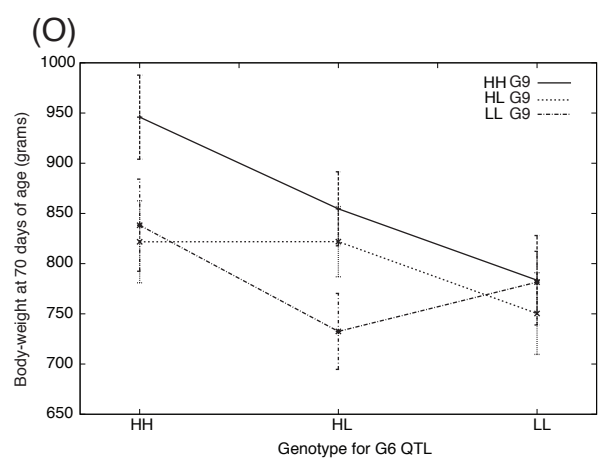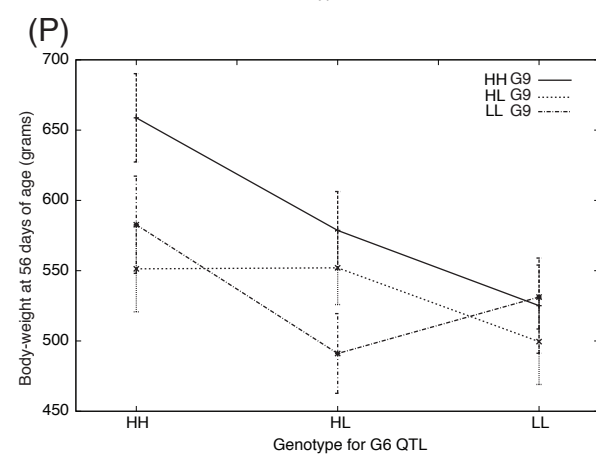

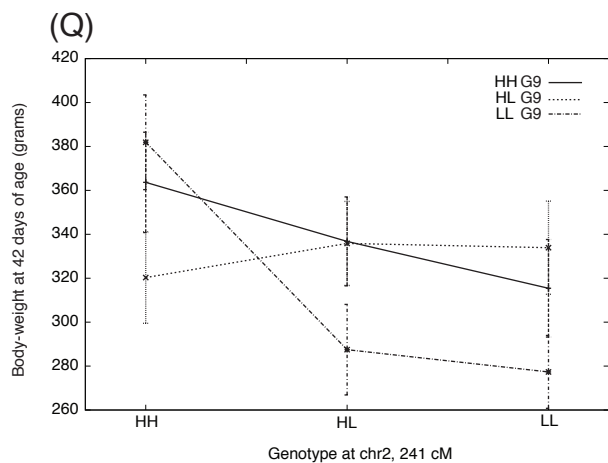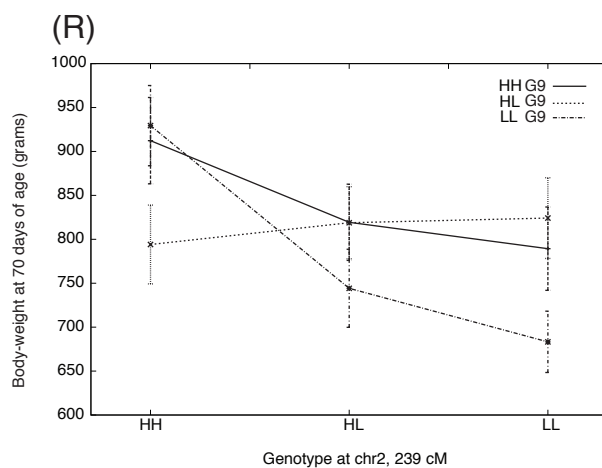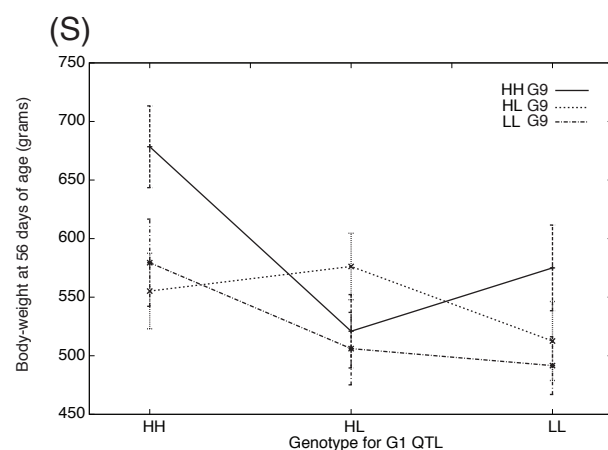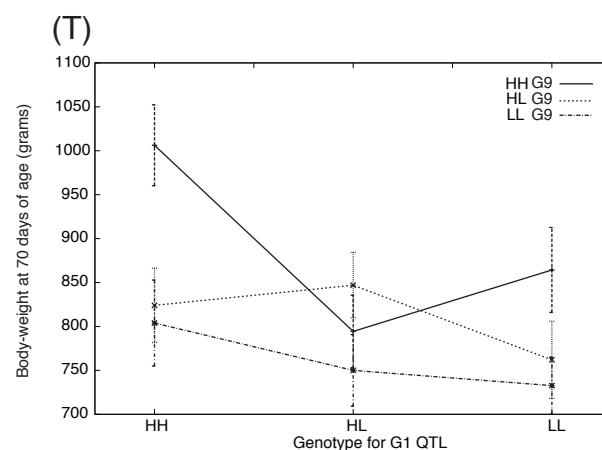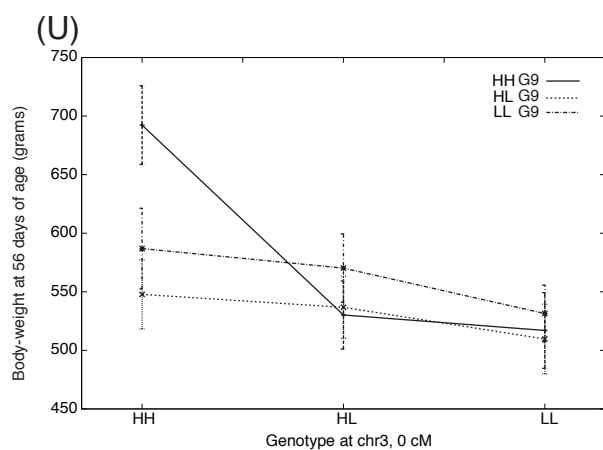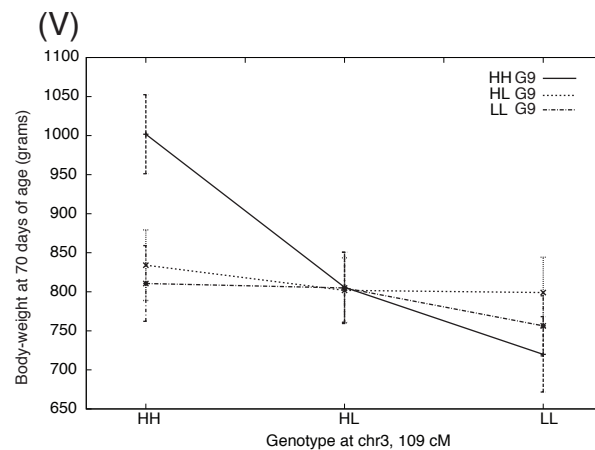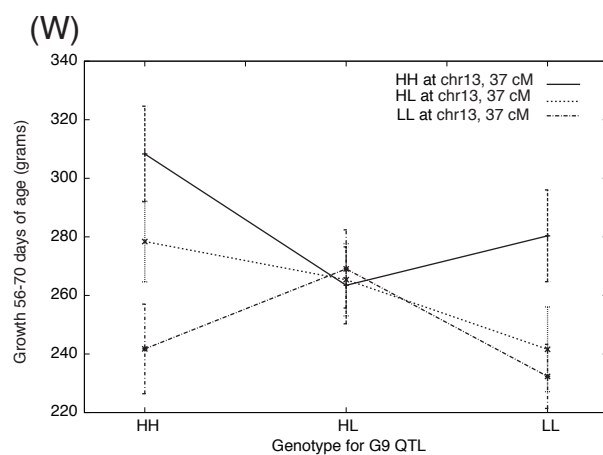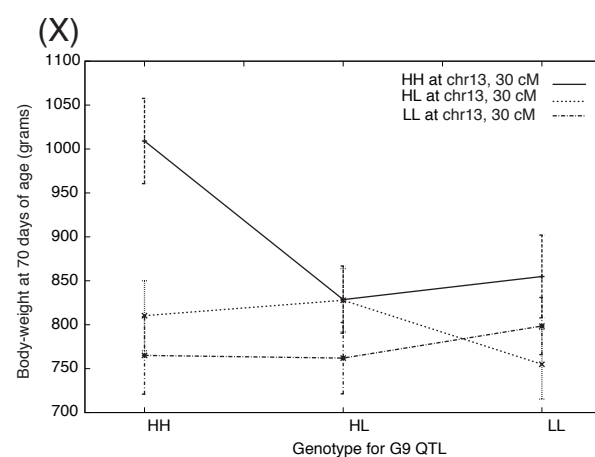

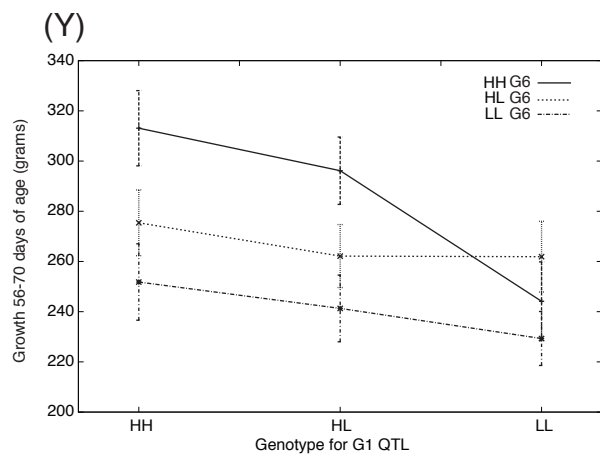

Supplement: Additional file 2 — Genotype-phenotype interaction maps for all epistatic QTL detected in the intercross for growth traits. Mean values of body-weight (in grams) for each of nine possible allelic combinations are shown in the plots. The genotype classes for one QTL is given on the X-axis (i.e. HH, HL and LL) and the curves represent the genotype classes of the other QTL. Error bars represent the standard error of mean (s.e.m.). HWS alleles are abbreviated as "H" and LWS alleles as "L". The figure is arranged so that all traits significant for the same unique interaction pair are displayed together. (A) QTL at Chr1, 316 cM – G6, (B) QTL at Chr1, 565 cM – Chr2, 224 cM, (C-D) Chr1, 473 cM – Chr10, 64 cM, (E-F) G12 – Chr1, 477 cM, (G) G9 – Chr24, 49 cM, (H) G9 – Chr28, 11 cM, (I-K) G9 – G12, (L) G1 – G12, (M-P) G9-G6, (Q – R) G9 – Chr2, 240 cM, (S-T) G9 – G1, (U-V) G9 – Chr3, 0 cM, (W-X) G9 – chr13, 30 cM, (Y) G1 – G6. [file 1471-2164-10-248-S2.pdf]
